# Polyglycolic Acid Felt Sealing Method for Prevention of Bleeding Related to Endoscopic Submucosal Dissection in Patients Taking Antithrombotic Agents

**DOI:** 10.1155/2016/1457357

**Published:** 2016-02-28

**Authors:** Hiroko Fukuda, Naoyuki Yamaguchi, Hajime Isomoto, Kayoko Matsushima, Hitomi Minami, Yuko Akazawa, Ken Ohnita, Fuminao Takeshima, Saburo Shikuwa, Kazuhiko Nakao

**Affiliations:** ^1^Department of Gastroenterology and Hepatology, Nagasaki University Hospital, 1-7-1 Sakamoto, Nagasaki-shi, Nagasaki 852-8501, Japan; ^2^Nagasaki University Graduate School of Biomedical Sciences, 1-7-1 Sakamoto, Nagasaki-shi, Nagasaki 852-8501, Japan; ^3^Department of Endoscopy, Nagasaki University Hospital, 1-7-1 Sakamoto, Nagasaki-shi, Nagasaki 852-8501, Japan; ^4^Division of Medicine and Clinical Science, Tottori University Faculty of Medicine, 36-1 Nishimachi, Yonago, Tottori 683-8504, Japan

## Abstract

*Background and Study Aims*. When performing endoscopic submucosal dissection (ESD) for patients on antithrombotic agents, the frequency of delayed bleeding is expected to increase. The endoscopic polyglycolic acid (PGA) felt and fibrin glue sealing method could be a new method for prevention of delayed bleeding.* Patients and Methods*. The safety and efficacy of the endoscopic tissue sealing method with PGA sheets and fibrin glue for the prevention of post-ESD bleeding were examined in 104 patients taking antithrombotic agents. During the study period, 70 patients taking antithrombotic agents did not undergo the sealing method, 36 patients discontinued antithrombotic agents, and 724 patients had not received antithrombotic therapy.* Results*. Delayed bleeding rates were 3.8% (4/104) in the sealing group, 12.9% (9/70) in the nonsealing group, 8.3% (3/36) in the discontinuation group, and 4.6% (33/724) in the nonantithrombotic therapy group. Thus, the delayed bleeding rate was significantly lower in the sealing group than in the nonsealing group and comparable to that in the nonantithrombotic therapy group.* Conclusions*. This PGA felt and fibrin glue sealing method might become a promising post-ESD bleeding prevention method in patients taking antithrombotic agents (UMIN000013990, UMIN000013993).

## 1. Introduction

With the advent of a superaging society, the number of patients taking antithrombotic agents is increasing. A gastrointestinal endoscopy practice guideline for antithrombotic agent recipients was established in 2012 [1]. This shows that one must consider the induction of thromboembolism by washout of the antithrombotic agents, as well as gastrointestinal bleeding due to continued antithrombotic agents.

However, it has been reported that the postoperative bleeding rate after gastric endoscopic submucosal dissection (ESD) is high in those who continue oral antiplatelet agents or are on anticoagulant agents and alternative therapy with heparin [2–6]. Therefore, a new bleeding prevention method is necessary after ESD for patients on antithrombotic agents.

There is a possibility that the endoscopic polyglycolic acid (PGA) felt and fibrin glue sealing method, which Takimoto et al. used for the first time to prevent delayed perforation after duodenal ESD [7], could be a new method for the prevention of delayed bleeding. Based on this background, the safety and efficacy of the endoscopic tissue sealing method with PGA felt and fibrin glue for prevention of post-ESD bleeding in patients taking antiplatelet agents or anticoagulation agents and alternative therapy with heparin were evaluated.

## 2. Methods

This was a retrospective, single-center analysis. This nonrandomized trial was undertaken at the Nagasaki University Hospital, Nagasaki, Japan. The study protocol was approved by the research ethics committee of the Nagasaki University Hospital on July 25, 2013, and was registered in the University Hospital Medical Network Clinical Trial Registry (UMIN-CTR) on June 1, 2014 (UMIN000013990, UMIN000013993).

### 2.1. Patients

The start time of the study was set during July 2012 when the new Japan Gastroenterological Endoscopy Society guidelines [1] were revised. The first sealing group patient was enrolled after study registration, and all sealing group patients gave their written informed consent for the intervention and for ESD. In the other group, patient consent was waived, given the retrospective nature of this study.

A total of 934 cases that underwent ESD in our hospital from July 2012 to December 2015 were enrolled in this study. They were classified into the following 4 groups. The sealing group included patients who continued antiplatelet agents or discontinued anticoagulation agents and alternative therapy with heparin and underwent the PGA sheets + fibrin glue sealing method, and the nonsealing group included patients who did not undergo the sealing method. The discontinuation group included patients who discontinued antithrombotic agents and did not undergo the sealing method. Continuation or cessation of antithrombotics was decided according to the new guidelines [1]. This was a nonrandomized study, so, basically, the sealing group was performed for all patients who continued antithrombotic agents after study registration. There were 104 enrolled cases in the sealing group, 70 in the nonsealing group, 36 in the discontinuation group, and 724 in the nonantithrombotic therapy group during this study period.

### 2.2. Definition of Antithrombotics and Management of Drugs before and after ESD

Antiplatelet agents or anticoagulants were defined according to the new guidelines [1].

In the sealing group and the nonsealing group, a single antiplatelet drug was continued during treatment. The patients who were prescribed a combination of drugs such as thienopyridine and low-dose aspirin (LDA) had 5–7 days of cessation except for LDA. The anticoagulant warfarin was discontinued 3–5 days before, and continuous infusion of intravenous undifferentiated heparin was administered at a dosage of 10,000–20,000 units per day. Heparin was stopped 6 hours before the ESD, and heparin was resumed on the day after ESD, after hemostasis had been confirmed. Oral warfarin was then resumed when oral ingestion was started. Novel oral anticoagulants (NOACs) were stopped 24 to 48 hours before ESD, and similarly, alternative therapy with heparin was performed, and NOACs were resumed on the day after ESD after hemostasis had been confirmed.

### 2.3. Endoscopic PGA Felt (Neoveil®, Gunze Co., Tokyo, Japan) + Fibrin Glue Sealing Method (Figures 1 and 2)

PGA felt 10 cm × 5 cm × 0.15 mm, cut to a size of 2 cm × 1.5 cm (2 cm group) or 5 cm × 5 cm (5 cm group), was used, and the pieces were attached using endoscopic forceps onto the ulcer surface just after ESD. In the case of the 5 cm group, modified procedures reported by Ono et al. [8] were used, and PGA felt was fastened with clips at the edges of the post-ESD ulcer. Then, using fibrin glue solutions (2-3 mL), the PGA felt pieces were adhered onto the ulcer surface. The fibrin glue consisted of solution A (fibrinogen) and solution B (thrombin); these were dropped in turn using the spraying tube onto the post-ESD ulcer surface after attaching the PGA felt, fibrin glue was formed, and the PGA felt was strongly bonded for a long period of time.

### 2.4. Evaluation Method

For the four groups, the following four items were assessed for each:

1. Treatment outcome (complete resection rate, curative resection rate, and en bloc resection rate).
2. Complications (control of intraoperative bleeding, delayed bleeding, and perforation).
3. Retention period of PGA felt.
4. Bleeding rate and retention period classified by PGA felt size.

*Evaluation Criteria*. The criteria for intraoperative bleeding control failure were the presence of intraoperative bleeding and hemoglobin (Hb) values decrease by ≥2 g/dL after treatment. Criteria for delayed bleeding were the development of hematemesis or hemorrhagic stool, Hb values decrease by ≥2 g/dL, and requiring endoscopic hemostasis. In cases of esophageal and gastric ESD, the criterion for the retention period of PGA was the period during which ≥50% of the adhered ulcer surface was covered. In cases of colonic ESD, where a 5 cm × 5 cm + clip fastening procedure was performed, the retention period was the time during which more than half of the fixing clips were left on abdominal X-ray.

Delayed bleeding was selected as the primary endpoint. Additionally, treatment outcome and other complications were examined. In the sealing group, the retention period of PGA felt and the bleeding rate or retention period classified by PGA felt size were examined.

### 2.5. Statistical Analysis

All statistical analyses were performed with StatFlex ver. 6.0 software (Artech Co., Ltd., Osaka, Japan). Categorical data were compared using *χ* ^2^ test or Fisher's exact test, as appropriate. The differences in the means of continuous data were compared by Student's *t*-test or the Mann-Whitney *U* test. *p* < 0.05 was considered significant. Bonferroni correction was performed for multiple comparisons.

## 3. Results

The baseline characteristics of the patients and tumors are listed in Table 1, and treatment outcomes are shown in Table 2. Age was significantly less in the nonantithrombotic therapy group than in the three other groups (*p* < 0.001). There were no significant differences among the four groups in the treatment outcomes.

All group complications are shown in Table 3. The 934 cases that underwent ESD during the study period in our hospital were classified into the sealing group (104 cases), the nonsealing group (70 cases), the discontinuation group (36 cases), and the nonantithrombotic therapy group (724 cases). The delayed bleeding rate was 3.8% (4/104) in the sealing group, 12.9% in the nonsealing group, and 4.6% in the nonantithrombotic therapy group; it was significantly lower in the sealing group than in the nonsealing group and was equivalent to that in the nonantithrombotic therapy group.  Table 4 shows delayed bleeding rate by organ. The delayed bleeding rate in the sealing group was significantly lower than in the nonsealing group only in the colon cases. However, in esophagus and gastric cases, there are no significant differences among the four groups.

Otherwise, there were no significant differences in the intraoperative bleeding control rate and the perforation rate.

*Retention Period of the PGA Felt*. The mean overall retention period of the PGA felt was 12.7 days (range: 1–32 days). 

*Bleeding Rate and Retention Period Classified by PGA Felt Size (Tables 5 and 6)*. In the sealing group, delayed bleeding was assessed according to PGA felt size. The delayed bleeding rate was 2.4% (2/84) in the 2 cm group and 10.0% (2/20) in the 5 cm group; thus, it was not significantly different, but it tended to be lower in the 2 cm group. Moreover, the mean retention periods were 13.7 and 9.8 days for the 2 cm and 5 cm groups, respectively. The retention period was significantly longer (*p* < 0.05) in the 2 cm group.

## 4. Discussion

The Japan gastrointestinal endoscopy practice guideline for antithrombotic agent recipients was established in 2012 [1]. It notes that it is important to consider the induction of thromboembolism by washout of the antithrombotic agents, as well as gastrointestinal bleeding due to continued antithrombotic agents. After revision of the guideline, there have been some reports about characteristics or management in endoscopy for patients with antithrombotic agents [9–11]. ESD has been accepted, because of its minimal invasiveness and high curative effect. However, complications such as bleeding are still an important problem.

It is known that such embolism risks as cardiovascular events and cerebral infarction are increased about three times by the discontinuation of aspirin, and in 70% of the patients, the events develop within 10 days after withdrawal [12, 13]. This may correspond to the period when hemorrhagic problems may occur just following endoscopic therapy. It has also been reported that discontinuation of anticoagulants in the perioperative period could cause embolism and thrombosis at a frequency of about 1%, possibly linked to poor clinical outcomes [14–16]. Therefore, in the new guideline, when endoscopists perform endoscopic therapies with a high risk of hemorrhage, such as ESD, anticoagulants are discontinued, and alternative therapy with heparin has been recommended in the perioperative period.

On the other hand, there is a prior report that there was no difference in the incidence of postoperative hemorrhage in the aspirin discontinued and continued groups in gastric ESD [2, 4]. However, in other reports, hemorrhage rates ranged from 11.6% to 31.8% when the antiplatelet agents were continued during ESD [2, 3]; the rates tended to be higher than in the nonantithrombotic therapy group (5.2% to 6.6%) and the discontinuation group (5.9% to 12.1%) [2–4]. Furthermore, the bleeding rate in patients who were given two or more antithrombotic agents was very high (46.7%), half of which led to massive bleeding requiring blood transfusion [3]. The hemorrhage rate after the anticoagulant heparin substitution method in gastric ESD was significantly higher (40.0%) than in the nonsubstitution group (4.2%) and the nonantithrombotic therapy group (5.2%) [5, 6]. In addition, hemorrhage rates in patients who had colorectal EMR or polypectomy during ongoing anticoagulant treatment ranged from 3% to 11%, which tended to be higher compared to about 1% in those without anticoagulant treatment. In particular, it has been reported that anticoagulant heparin substitution led to gastrointestinal bleeding in 18% of cases, which tended to be higher than the 3.1% in patients who did not receive heparin substitution [6, 17, 18].

That is, in patients with a risk of thrombosis requiring oral antithrombotic agents, discontinuation of the antithrombotic agents would be linked to increased thrombosis risk. However, on the other hand, continuation of the agents would increase the hemorrhage risk during and following ESD. In order to overcome this dilemma, a strategy for hemorrhage prevention related to ESD is required in cases of antiplatelet agent continuation or anticoagulant heparin substitution.

PGA felt is an artificial fiber sheet that is biocompatible and spontaneously absorbable over about 3 months. The coating method in combination with fibrin glue and PGA felt has been widely used clinically for diverse purposes, such as prevention of scar stenosis or air leaks in thoracic surgery, otolaryngology, neurosurgery, and so on. More recently, in gastrointestinal surgical procedures, it has been found to substantially reduce postoperative bleeding by coating the resection surface following pancreatectomy or hepatectomy. Therefore, there is a possibility that the endoscopic PGA felt and fibrin glue sealing method which Takimoto et al. used to prevent delayed perforation after duodenal ESD [7] could be a treatment of choice as a new method for prevention of delayed bleeding. In the endoscopic therapy areas, this coating method provides a scaffold for tissue repair regeneration of the ulcer surface, and it can protect it from the physical stimulation of food and the chemical stimulation of digestive enzymes such as gastric acid, bile, and pancreatic juice. There is an effect to promote ulcer healing, and it can be expected to prevent delayed perforation, as well as hemorrhage, after ESD. It was reported that, with this method, there was less inflammatory cell infiltration on histopathology than in controls in an animal experiment [19, 20]. Tsuji et al. reported that PGA felt sealing method might prevent post-ESD bleeding for high-risk group, such as large mucosal resection or use of antithrombotic drugs [21]. Thus, we examined the safety and efficacy of the sealing method for prevention of post-ESD bleeding in patients taking antithrombotic agents.

In the present study, 934 cases underwent ESD during the same period in our hospital. A total of 174 ESDs were performed for patients continuing antiplatelet agents or anticoagulation agents and alternative therapy with heparin, and of these, the PGA felt and fibrin glue sealing method was used in 104. The delayed bleeding rate was significantly lower in the sealing group than in the nonsealing group and was equivalent to that in the nonantithrombotic therapy group. Otherwise, there were no significant differences in the complete resection rate, curative resection rate, intraoperative bleeding control rate, and perforation rate. Furthermore, the mean retention period of PGA felt was 12.7 days, and it corresponded to the 5–8 days when delayed bleeding might occur in relation to ESD. When compared according to PGA felt size, the PGA felt remained significantly longer in the 2 cm group, and the delayed bleeding rate was low. It appears that 2 cm felt is more useful for prevention of delayed bleeding.

These results suggest that this PGA felt and fibrin glue sealing method might become a new post-ESD bleeding prevention method in patients continuing antithrombotic agents or those given anticoagulant heparin substitution.

However, the four bleeding cases in the sealing group were all gastric ESD cases. Considering only gastric cases, the delayed bleeding rate had no significant difference among the four groups. In all four bleeding cases, PGA felt dropped out early and had already dropped at the time of the bleeding. Accordingly, it is thought that it is important that the PGA felt remain for the long term.

Furthermore, this study had limitations because of the small number of cases and its design as a retrospective, uncontrolled study. Further studies are warranted to establish the safety of ESD, including the delayed bleeding prevention effect in patients continuing antiplatelet agents or anticoagulation agents and alternative therapy with heparin.

## 5. Conclusion

The PGA felt and fibrin glue sealing method might become a new post-ESD bleeding prevention method in patients continuing antithrombotic agents or anticoagulant heparin substitution.

## Figures and Tables

**Figure 1:**
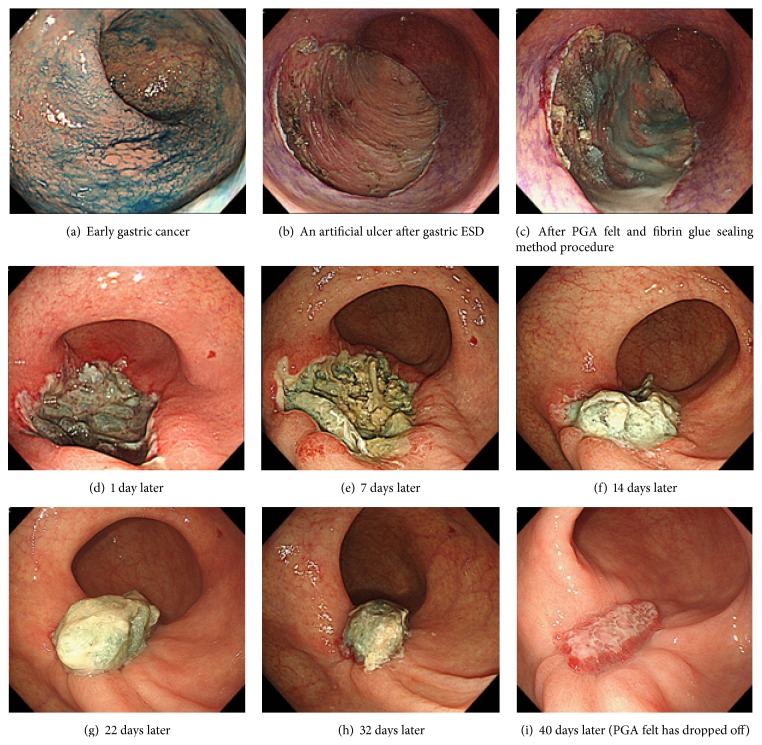
Case 1: endoscopic view of ESD of an 84-year-old woman with early gastric cancer under continued antiplatelet agents (LDA) and PGA felt (2 cm × 1.5 cm) and fibrin glue sealing method.

**Figure 2:**
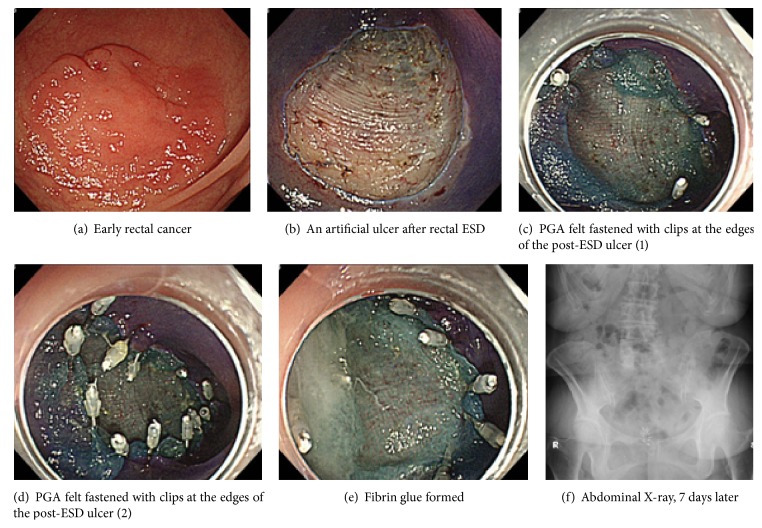
Case 2: endoscopic view of ESD of a 73-year-old woman with early rectal cancer under heparin replacement and PGA felt (5 cm × 5 cm) and fibrin glue sealing method.

**Table 1:** Baseline characteristics of patients and tumors.

	Sealing group (*n* = 104)	Nonsealing group (*n* = 70)	Discontinuation group (*n* = 36)	Nonantithrombotic therapy group (*n* = 724)
Mean age (years)	74.8 ± 7.1	75.2 ± 9.2	74.8 ± 6.9	68.9 ± 9.8
Sex (males/females)	76/28	52/18	27/9	504/220
Tumor location (E/S/C)^*∗*^	28/48/28	11/44/15	5/23/8	206/343/175
Mean resection size (mm)	43.4 ± 13.4	45.3 ± 14.5	43.8 ± 16.8	44.5 ± 16.3
Antithrombotics				—
Antiplatelets	86	45	35	
Low dose aspirin only	53	28	12	
Thienopyridine only	10	4	0	
DAPT^*∗*^	10	4	4	
Anticoagulants	19	26	1	
NOAC^*∗*^	9	2	1	

^*∗*^E/S/C: esophagus/stomach/colon; DAPT: dual antiplatelet therapy; NOAC: novel oral anticoagulant.

**Table 2:** Treatment outcomes.

	Sealing group (*n* = 111)	Nonsealing group (*n* = 74)	Discontinuation group (*n* = 37)	Nonantithrombotic therapy group (*n* = 761)	*p* value
Complete resection rate	95.5% (106/111)	98.6% (73/74)	100% (37/37)	95.8% (729/761)	n.s.
Curative resection rate	90.1% (100/111)	93.2% (69/74)	94.6% (35/37)	88.0% (670/761)	n.s.
*En bloc* resection rate	99.1% (110/111)	100% (74/74)	100% (37/37)	99.5% (757/761)	n.s.

**Table 3:** Complications.

	Sealing group (*n* = 104)	Nonsealing group (*n* = 70)	Discontinuation group (*n* = 36)	Nonantithrombotic therapy group (*n* = 724)	*p* value
Perforation rate	1.0% (1/104)	2.9% (2/70)	2.8% (1/36)	2.9% (21/724)	n.s.
Intraoperative bleeding control failure rate	0% (0/104)	0% (0/70)	0% (0/36)	0.7% (5/724)	n.s.
Delayed bleeding rate	3.8% (4/104)^*∗*^	12.9% (9/70)^*∗*,*∗∗*^	8.3% (3/36)	4.6% (33/724)^*∗∗*^	^*∗*^<0.05 ^*∗∗*^<0.01

*∗* represents *p*-value of when comparing the sealing group and nonsealing group. *∗∗* represents *p*-value of when comparing the nonsealing group and nonantithrombotics group.

**Table 4:** Delayed bleeding rate by organ.

	Sealing group (*n* = 104)	Nonsealing group (*n* = 70)	Discontinuation group (*n* = 36)	Nonantithrombotic therapy group (*n* = 724)	*p* value
Esophagus	0.0% (0/28)	0.0% (0/11)	0.0% (0/5)	0.5% (1/206)	n.s.
Stomach	8.3% (4/48)	11.4% (5/44)	13.0% (3/23)	7.0% (24/343)	n.s.
Colon	0.0% (0/28)^*∗*^	26.7% (4/15)^*∗*,*∗∗*^	0.0% (0/8)	4.6% (8/175)^*∗∗*^	^*∗*^<0.01 ^*∗∗*^<0.01
Total	3.8% (4/104)^*∗*^	12.9% (9/70)^*∗*,*∗∗*^	8.3% (3/36)	4.6% (33/724)^*∗∗*^	^*∗*^<0.05 ^*∗∗*^<0.01

*∗* represents *p*-value of when comparing the sealing group and nonsealing group. *∗∗* represents *p*-value of when comparing the nonsealing group and nonantithrombotics group.

**Table 5:** Bleeding rate and retention period classified by PGA felt size.

	2 cm group	5 cm group
Esophagus	0.0% (0/25)	0.0% (0/3)
Stomach	5.1% (2/39)	22.2% (2/9)
Colon	0.0% (0/20)	0.0% (0/8)
Total	2.4% (2/84)	10.0% (2/20)

**Table 6:** Retention period classified by PGA felt size.

	2 cm group	5 cm group	
Esophagus	11.2 ± 8.7 days (1–28 days)	7.0 ± 0.0 days (7 days)	
Stomach	15.3 ± 7.4 days (1–32 days)	11.2 ± 6.8 days (1–25 days)	
Colon	—	9.3 ± 5.0 days (7–21 days)	
Total	13.7 ± 8.1 days (1–32 days)	9.8 ± 5.6 days (1–25 days)	*p* < 0.05
